# Transcriptome response to heat stress in a chicken hepatocellular carcinoma cell line

**DOI:** 10.1007/s12192-015-0621-0

**Published:** 2015-08-05

**Authors:** Liang Sun, Susan J. Lamont, Amanda M. Cooksey, Fiona McCarthy, Catalina O. Tudor, K. Vijay-Shanker, Rachael M. DeRita, Max Rothschild, Chris Ashwell, Michael E. Persia, Carl J. Schmidt

**Affiliations:** Department of Animal and Food Sciences, University of Delaware, Newark, DE 19716 USA; Department of Animal Science, Iowa State University, Ames, IA 50011 USA; School of Animal and Comparative Biomedical Sciences, University of Arizona, Tucson, AZ 85721 USA; Department of Computer and Information Sciences, University of Delaware, Newark, DE 19716 USA; Department of Poultry Science, North Carolina State University, Raleigh, NC 27695 USA

**Keywords:** Transcriptome, RNA-seq, Heat stress, Chicken, LMH cells

## Abstract

Heat stress triggers an evolutionarily conserved set of responses in cells. The transcriptome responds to hyperthermia by altering expression of genes to adapt the cell or organism to survive the heat challenge. RNA-seq technology allows rapid identification of environmentally responsive genes on a large scale. In this study, we have used RNA-seq to identify heat stress responsive genes in the chicken male white leghorn hepatocellular (LMH) cell line. The transcripts of 812 genes were responsive to heat stress (*p* < 0.01) with 235 genes upregulated and 577 downregulated following 2.5 h of heat stress. Among the upregulated were genes whose products function as chaperones, along with genes affecting collagen synthesis and deposition, transcription factors, chromatin remodelers, and genes modulating the WNT and TGF-beta pathways. Predominant among the downregulated genes were ones that affect DNA replication and repair along with chromosomal segregation. Many of the genes identified in this study have not been previously implicated in the heat stress response. These data extend our understanding of the transcriptome response to heat stress with many of the identified biological processes and pathways likely to function in adapting cells and organisms to hyperthermic stress. Furthermore, this study should provide important insight to future efforts attempting to improve species abilities to withstand heat stress through genome-wide association studies and breeding.

**Electronic supplementary material:**

The online version of this article (doi:10.1007/s12192-015-0621-0) contains supplementary material, which is available to authorized users.

## Introduction

Fluctuations in environmental temperatures are encountered over the life span of most organisms. Many species have a metabolism that is adapted to the temperature range of the environment in which they evolved. When the external temperature rises above this range, the heat produced by a homothermic animal exceeds the amount that can be lost to the environment, resulting in a rise in body temperature. This triggers an evolutionarily conserved heat stress transcriptome response modulating genes that control multiple cellular activities including protein folding, protein degradation, transport, metabolism, DNA repair, and replication (Lindquist and Craig 1988; Feder and Hofmann 1999; Kregel 2002).

A starting point for understanding the heat stress response is identifying the genes that are modulated by hyperthermia. Numerous studies have identified genes that respond to heat stress, but high-throughput transcriptome sequencing is a technology able to provide a more complete catalog of heat responsive genes (Szustakowski et al. 2007; Li et al. 2011; Islam et al. 2013; Kristiansson et al. 2013; Smith et al. 2013; Wang et al. 2013). While the ultimate goal might be to identify every gene in every tissue of an organism that responds to heat stress, a practical first step is to use in vitro cell culture methods. Cultured cells can be raised under defined temperature conditions for precise amounts of time, allowing for careful control of their environment. Subjecting a cell line to heat stress and comparing the transcriptome results with control cells will identify heat responsive genes. The objective of this work was to use transcriptome sequencing to define the heat stress response of the chicken hepatocellular carcinoma cell line, LMH (Kawaguchi et al. 1987), which was developed from a male white leghorn chicken. A total of 812 genes consistently responded to heat stress with 235 induced and 577 repressed following treatment. Enrichment analysis identified functional groupings such as molecular chaperones and transcription factors within the induced genes, and DNA replication and DNA repair among the repressed genes.

## Materials and methods

### Cell culture

LMH cells were obtained from ATCC (Manassas, VA) and cultured in Waymouth’s MB medium with 10 % heat-inactivated fetal bovine serum in flasks coated with 0.1 % gelatin. Cells were cultured at 37 °C in 5 % CO_2_ and passaged every 2–3 days. Prior to heat stress, cells were grown to 80 % confluence. Three T-75 (Falcon) flasks were maintained at control temperature and a second set of three flasks subjected to heat challenge. Six individual flasks of control cells were cultured at 37 °C while six flasks of heat-stressed cells were maintained at 43 °C for 2.5 h. This protocol was chosen as prior published work indicated it would provide for maximal heat stress response (Gabis et al. 1996).

### RNA preparation

Cells were released from flasks with trypsin and collected in 50-ml conical tubes by centrifugation (1200 RPM; at 37 °C for control cells, 43 °C for heat-stressed cells). RNA was prepared using the Qiagen RNeasy mini kit with DNAase treatment and quality-checked using the Agilent 2100 Bioanalyzer. RNA with a RIN value greater than 9.0 was used for transcriptome library production.

### Transcriptome production and data analyses

Transcriptome libraries were prepared separately from each control and experimental flask using the Illumina TrueSeq RNA Sample Preparation Kit. The 12 individual libraries were sequenced on an Illumina HiSeq 2500 at the University of Delaware DNA Sequencing Facility. Sequences were mapped to the 2006 release of the Red Jungle Fowl genome (International Chicken Genome Sequencing, C 2004) and gene Reads Per Kilobase per Million (RPKM) values assigned using ERANGE (Mortazavi et al. 2008). Expression data were statistically analyzed using JMP, and genes were assigned to functional groups using DAVID (Huang et al. 2009a, 2009b) and eGIFT (Tudor et al. 2010). All fastq files have been submitted to the GEO database under GEO number GSE55321.

### Quantitative RT-PCR

First-strand synthesis was done with superscript II Reverse Transcriptase (Life Technologies) according to the manufacturer’s directions. Quantitative reverse transcription PCR (qRT-PCR) was performed using gene-specific primers (Table 2) and the Fast SYBER green master mix (Applied Biosystems) on an Applied Biosystems 7500 Fast Real Time PCR System according to the manufacturer’s directions.

## Results and discussion

### Overview of results

**Fig. 1:**
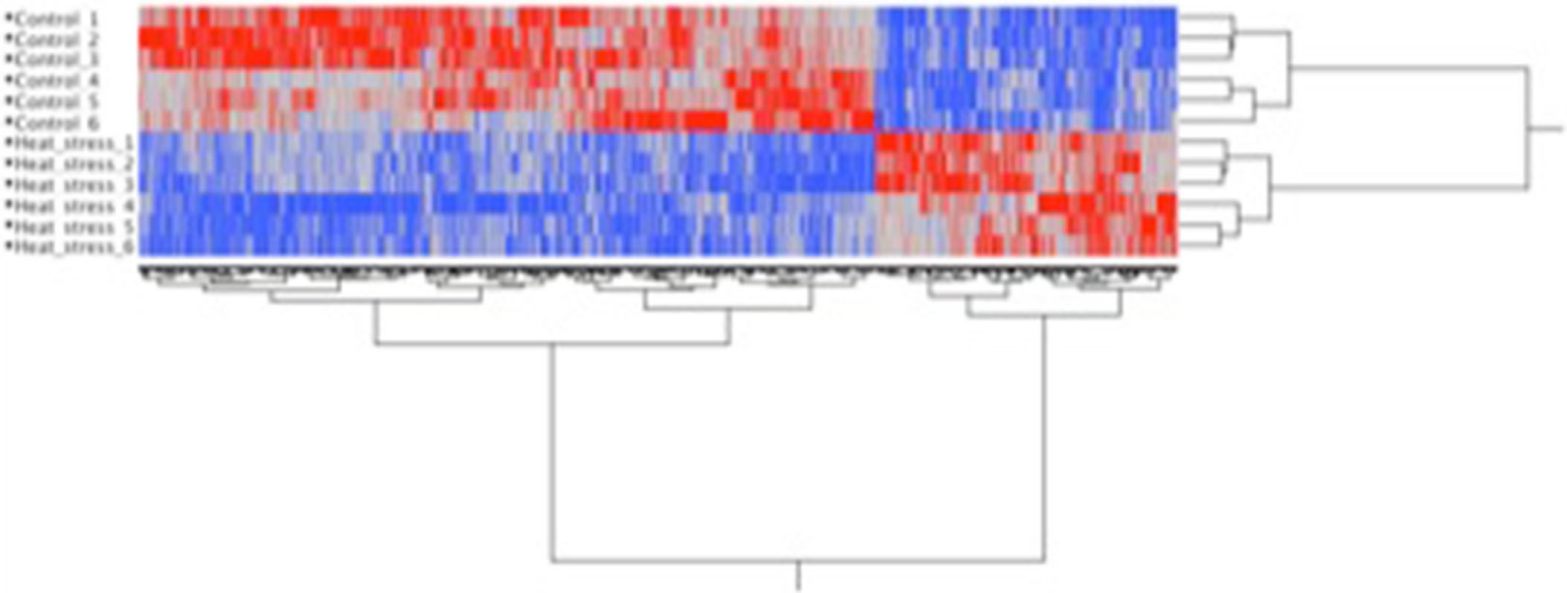
Hierarchical clustering Hierarchical cluster analysis of transcriptome data from heat challenged and control cells. Note that the control and heat stress samples cluster separately. Red corresponds to genes whose transcripts were enriched in the corresponding sample while blue corresponds to genes whose transcripts were reduced in that sample.

Over 250 million transcriptome reads were generated across 12 separate flasks of control or heat-stressed LMH cells (Table 1). A total of 15,945 chicken genes were analyzed in LMH cells for their response to heat stress at 43 °C versus 37 °C for 2.5 h (Supplementary File 1). A total of 12,299 genes were detected under heat or control conditions with RPKM values greater than 0.1. The log_2_ values of the ratio between the heat stress and control RPKM were determined for each of these genes. A *t* test was applied to identify genes that were significantly differentially expressed between the two conditions (*p* < 0.01). This generated a list of 812 genes that reproducibly responded to heat challenge across all 12 samples, with 235 genes whose expression was increased and 577 genes whose expression was decreased after heat challenge (Supplementary File 2). Hierarchical clustering (Fig. 1) of the 812 responsive genes segregated the samples based on environmental condition (heat vs. control) with principal component analysis (Supplementary File 3) indicating that 22 % of the variance in the data was associated with the cell incubation temperature. For a complete list of all genes discussed in this paper, see Supplementary File 4.

**Table 1:** Transcriptome Read Depth for Each Sample Library

Library	Total Reads	Mapped Reads	Unique Reads
Control 1	26,481,906	89.9 %	95.9 %
Control 2	35,224,137	89.8 %	96.4 %
Control 3	35,841,457	90.4 %	95.8 %
Control 4	22,491,917	87.8 %	96.0 %
Control 5	17,147,757	87.3 %	96.5 %
Control 6	13,106,307	87.4 %	96.6 %
Heat stress 1	33,816,428	90.0 %	95.2 %
Heat stress 2	13,710,771	90.0 %	95.8 %
Heat stress 3	27,526,906	90.4 %	96.0 %
Heat stress 4	19,754,286	87.6 %	96.2 %
Heat stress 5	26,463,555	87.7 %	96.1 %
Heat stress 6	31,610,408	88.2 %	96.2 %

The total number of sequences from each library prepared from separate flasks of either control (37 °C) or heat stressed (43 °C) LMH cells is given in the Total Reads column. The Mapped Reads column is the percentage of these total reads that mapped to the chicken genome. The Unique Reads column is the percentage of mapped reads that only correspond to one site in the genome. Combined, this data yielded more than 25 × 10^7^ reads that mapped uniquely to the chicken genome.

### Validation

**Fig. 2:**
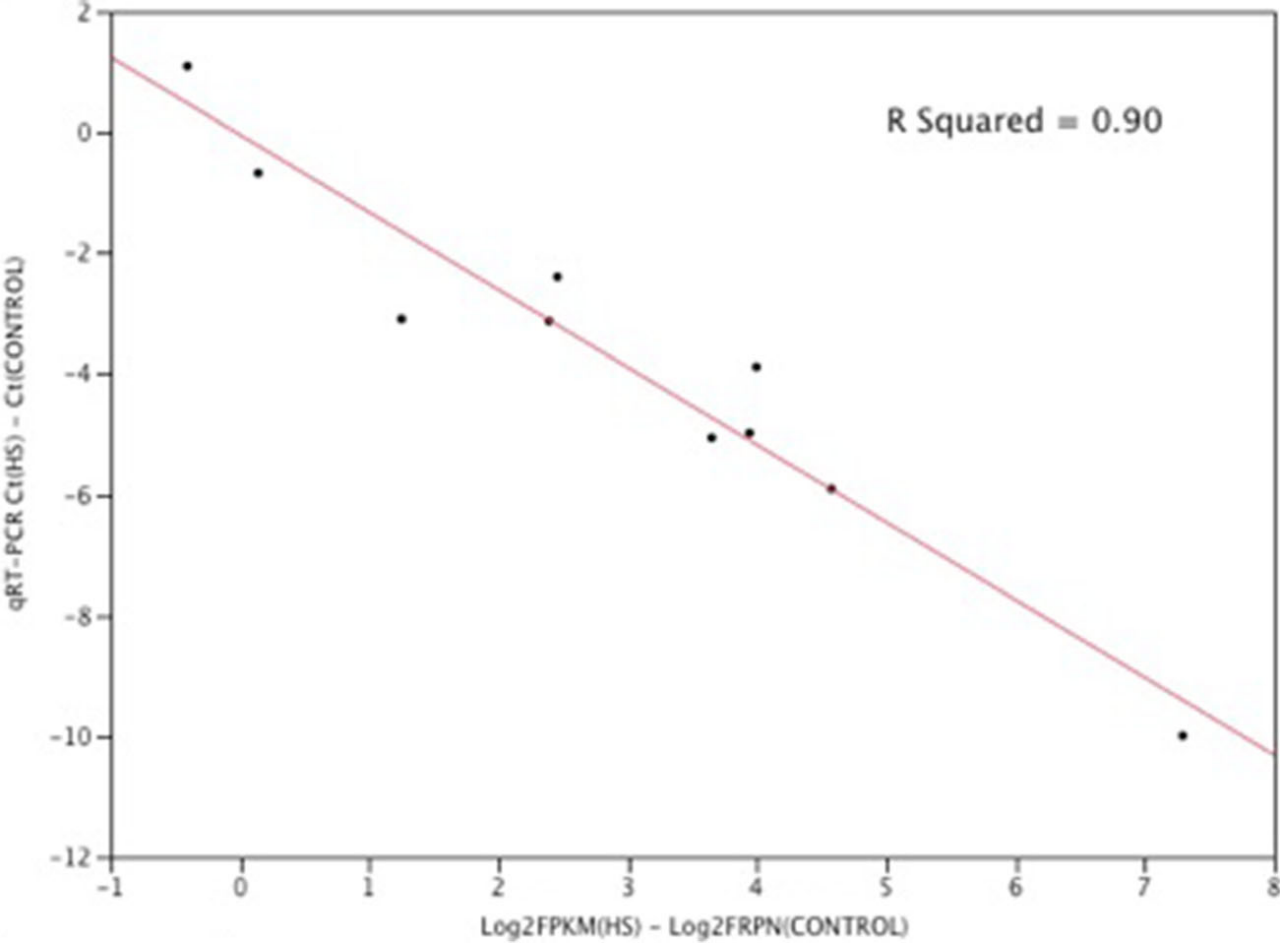
Relationship between RNA-seq and qRT-PCR R-squared analysis of the fold difference relationship between heat challenged and control levels in the expression of 10 genes as determined by transcriptome analysis (X-axis) and quantitative reverse transcriptase PCR (Y-axis).

Illumina RNA-seq transcriptome data was validated by quantitative RT-PCR (qRT-PCR) using ten genes, comparing expression levels between control and heat-stressed samples (Table 2). Although the absolute fold changes differed between qRT-PCR and RNA-seq, the direction of change was concordant for each gene. Plotting the qRT-PCR delta Ct values (difference between heat stress and control Ct values) versus the Log_2_RPKM for the genes yielded an *R*^2^ value of 0.90 (Fig. 2), which is consistent with other studies comparing correlations between RNA-seq and qRT-PCR studies (Core et al. 2008; Nagalakshmi et al. 2008; Camarena et al. 2010; Feng et al. 2010).

**Table 2:** Gene symbols and primers used for qRT-PCR validation of RNA seq dat

Gene	Primer (5′- > 3′)
BAG3_R	GATGGGAGTTGAGGGCTGTA
BAG3_F	TACCATCAGGCCCAGAAGAC
CAPS2_R	CCCCATGGGTTCCTTAAGAT
CAPS2_F	GGCAGGCAAAGCTACAGAAG
DNAJA4_R	TCTATTCATTCGGCCTCCAC
DNAJA4_F	GAAGTACCACCCCGACAAGA
GABRA2_R	TGAATTTCGAGCACTGATGC
GABRA2_F	GGCCAAACAATTGGAAAAGA
HMOX1_R	GACGCCGTGACCAGCTTGAAC
HMOX1_F	GCCACCAAGGAGGTGCACGA
HSP25_R	GGCGAAGTTCTTCACATCCT
HSP25_F	CACGCAGAGACCATCTTCAG
KLHDC2_R	GACGCCTTGTGCCATTATTT
KLHDC2_F	GCTCATGCGTGTGCTACAGT
MYCN_R	TTGGTTGGATCATGGGTTTT
MYCN_F	ACCACTTTTCCATCGGTCAG
P4HA2_R	CTGTGATCTGCTGCATTCGT
P4HA2_F	AACAACTGGCCAAACCAAAG
UCP3_R	GAACGACAAAGGTTGGCAGT
UCP3_F	CGGGATTTGATTCTGTGCTT

### Heat shock factors

Four heat shock factors (HSFs), HSF2, HSF3, HSF4, and HSF5, were detectably expressed in the LMH cells (Table 3). HSF2 and HSF3 transcripts were 30–100-fold more abundant than HSF4 and HSF5 although no HSF showed a significant change in expression as a function of temperature (Table 3). In the chicken, HSF1 is not yet mapped to a specific chromosome, and for this reason, we do not have a determined RPKM level for HSF1. However, inspection of the unmapped sequence reads by BLAST identifies chicken HSF1 in the LMH transcriptome. Hence, all five known HSFs are expressed in this cell line.

**Table 3:** RPKM values for Heat Shock Factor (HSF) transcription regulatory genes

Heat Shock Factor	RPKM Control	RPKM Heat Stress
HSF2	46.2	32.8
HSF3	8.9	10.7
HSF4	0.1	0.1
HSF5	0.5	0.3

### Chaperones, heat shock protein, and DNAJ genes

A major function of many heat shock inducible gene products is to serve as protein chaperones, assisting in folding of nascent proteins and inhibiting protein aggregation (Lindquist and Craig 1988; Hightower 1991; Moseley 1997). Fifteen genes encoding chaperones were modulated by heat stress (Table 4). The greatest induction was observed for LOC772158, a heat shock protein 30 (HSP30) like gene product (223-fold), while the least was DNAJB6 (2-fold). Two genes encoding products classified as chaperones, HSPA14 and DNAJC17, exhibited reduced expression following heat treatment.

**Table 4:** Fold change for Chaperone gene products

Entrez Gene ID	Symbol	Fold Change Heat Stress
772158	LOC772158	223
428310	HSP25	157.5
423504	HSPA2	64
416988	HSPB8	36.7
396228	SERPINH1	8.6
418917	HSPH1	8
423463	HSP90AA1	6.5
396487	HSPA5	5.3
395853	HSPA8	4
374163	HSP90B1	3.5
416339	HSPA4	3
427376	DNAJA1	3
420448	DNAJB6	2
770519	DNAJC17	0.4
418802	HSPA14	0.3

Fold change for Chaperone gene products as determined by: (Mean RPKM(43oC) ÷ Mean RPKM (37 °C))

DAVID functional enrichment analysis (Huang et al. 2009) of gene transcripts elevated by heat stress provided an overview of processes affected by hyperthermia. The functional groupings included Chaperone, Unfolded Protein Binding, Endoplasmic Reticulum, Protein Kinase Activity, and Regulation of Transcription. eGIFT (Tudor et al. 2010) identifies informative terms (iTerms) for individual genes using text-mining approaches. Because iTerms are not limited to ontology terms, iTerms can provide a finer grained interpretation of gene lists than GO ontology analysis (Table 5). The majority of iTerms refer to processes and functions typically modulated during heat stress including molecular chaperones, co-chaperone, endoplasmic reticulum (ER), secretion, and apoptosis. Many of the gene products in these groups are chaperones that assist in proper protein folding.

**Table 5:** iTERMS for gene significantly up-regulated by heat stress

iTERM	Gene symbols
SECRETION	HSP90B1, SFRP1, HSPA5, CYR61, NTNG1, WNT4, CAPS2, AMH, ABHD5, MYOC, SERPINH1, CTGF, CBLN1, SYT4, INHBA, ABCC2
APOPTOSIS	HSPA5, BAG3, HSPA4, LARP6, TIMP3, E2F7, HSP90AA1,KLF5, HSP25, ATF4, HSPH1, MAP3K5, TSC22D1, SQSTM1
TRANSCRIPTION FACTOR	ATF4, NFYA, RFX2, JDP2, NHLH1, CREM, PLAG1, TMEM173, NFYA, KLF5, KAT2A, SIK1, KLF6, NR0B1, TSC22D1
MOLECULAR CHAPERONES	HSP90B1, HSPA5, HSPA2, HSPA4, HSPA8, DNAJA1, HSP90AA1, SERPINH1, HSP25, HSPH1, DNAJB6, HSPB8
STRESS	HSP90B1, HSPA5, BAG3, HSP90AA1, SERPINH1 HSPA2, HSPA4, HSPA8, HSP25, ATF4, HSPH1

### Collagen scaffold

Six gene products affecting the collagen scaffold were elevated by heat stress: serpin peptidase inhibitor 1 (SERPINH1); La ribonucleoprotein domain family, member 6 (LARP6); TIMP metallopeptidase inhibitor 3 (TIMP3); discoidin domain receptor tyrosine kinase 2 (DDR2); connective tissue growth factor (CTGF); and cysteine-rich, angiogenic inducer, 61 (CYR61). SERPINH1 is a molecular chaperone responsible for proper folding and secretion of collagen (Kurkinen et al. 1984; Nagata et al. 1986; Cates et al. 1987) while LARP6 is a La ribonucleoprotein domain family member that binds the 5′ noncoding region of the collagen mRNA and directs collagen protein synthesis to discrete locations within the endoplasmic reticulum (Cai et al. 2010a, 2010b). TIMP3 (Pavloff et al. 1992) plays a role in the extracellular stabilization of collagen matrices by inhibiting metalloproteases (Leco et al. 2001), while DDR2 functions as a collagen receptor and plays an important role in suppressing liver fibrosis (Olaso et al. 2011). Finally, both CTGF and CYR61 are growth factors that, among other things, promote synthesis and deposition of collagen (Duncan et al. 1999; Brigstock 2002).

### Transcription factors

Several transcription factors were positively regulated by heat stress, two of which, activating transcription factor 4 (ATF4) and nuclear transcription factor A (NFYA), are known to couple ER stress to transcriptional regulation. Also enriched was the transcription factor E2F7 that regulates the cell cycle, with elevated E2F7 expression causing arrest in G2 phase (de Bruin et al. 2003). Fourteen other genes whose products regulate transcription showed a significant increase in expression when LMH cells were heat-stressed (Table 5). Salt-inducible kinase 1 (SIK1) responds to osmotic stress (Wang et al. 1999; Sjostrom et al. 2007), TSC22 domain family, member 1 (TSC22D1) is induced by TGFß (Kester et al. 1999), transmembrane protein 173 (TMEM173) responds to viral infection (Ishikawa et al. 2009), and regulatory factor X (RFX2) is involved in testes development (Horvath et al. 2004; Wolfe et al. 2004), but none of these have previously been shown to be heat responsive. Two members of the Kruppel-like transcription factor family, KLF5 and KLF6, also responded to heat stress in these experiments. To date, only KLF4 has been shown to be induced by heat stress (Liu et al. 2006); in our experiments, KLF4 expression was not detected in LMH cells. Perhaps the paralogous KLF5 and KLF6 gene products replace KLF4 function in LMH cells. Six additional transcription regulatory genes were enriched by heat challenge that have not, to our knowledge, been previously implicated in heat stress response including cAMP responsive element modulator (CREM), v-myc related oncogene, neuroblastoma derived (MYCN), nescient helix loop helix 1 (NHLH1), nuclear receptor subfamily 0, group B, member 1 (NR0B1), pleiomorphic adenoma gene 1 (PLAG1), and Scm-like with four MBT domains protein 1 (SFMBT1).

### Chromatin modification

Four gene products that were upregulated by heat stress control transcription by chromatin modification. K(lysine) acetyltransferase 2A (KAT2A) promotes histone acetylation (Nagy and Tora 2007), while Jun dimerization protein 2 (JDP2) inhibits (Jin et al. 2006) histone acetylation, jumonji domain containing 6 (JMJD6) is responsible for demethylation of HIS3 at arginine residues (Chang et al. 2007), and SFMBT1 is a member of the polycomb protein family that inhibits transcription by altering chromatin localization (Alfieri et al. 2013). Another four genes controlling epigenetic modulation are downregulated by heat stress: chromatin assembly factor 1, subunit B (CHAF1B); ubiquitin-like with PHD and ring finger domains 1 (UHRF1); DNA (cytosine-5-)-methyltransferase 3 beta (DNMT3B); and enhancer of zeste homolog 2 (EZH2). CHAF1B (Nabatiyan and Krude 2004), UHRF1 (Hashimoto et al. 2009), and DNMT3B (Wang et al. 2007) function in maintaining DNA methylation and silencing genes through incorporation of DNA into heterochromatin. EZH2 is a polycomb family member that is responsible for silencing genes during development by trimethylation of Histone H3 (Rajasekhar and Begemann 2007).

The different transcription factors and chromatin modifiers whose transcripts were affected by heat stress suggest a complex interplay between transcription activators, repressors, and epigenetic modifications in response to heat challenge. For example, KLF5 is a strong transcriptional activator typically expressed in proliferating cells of the gastrointestinal track that plays a role in suppressing apoptosis (Sun et al. 2001). In contrast, NR0B1 functions as a transcriptional repressor, possibly affecting the large number of genes whose expression is suppressed by heat stress in the LMH cells. At least eight genes affected by heat stress (up regulated or downregulated) modulate transcription by epigenetic mechanisms and could play an important role in both short- and long-term responses to heat. These epigenetic regulators control different types of modifications including DNA methylation, histone acetylation, and histone methylation. Exposure of chicken embryos to elevated temperatures during embryonic development improves the ability of the hatched bird to tolerate heat stress (Wang and Edens 1998; Pavani and Piestun 2008; Piestun et al. 2008, 2009; Willemsen et al. 2010; Al-Zhgoul et al. 2013). Conceivably, the epigenetic regulators identified in this study could be involved in inducing the tolerance phenomena.

### Signaling pathways

Two signal transduction cascades appear to be affected by heat stress: TGFß (Massague 1990; Lawrence 1996) and WNT (Clevers 2006). Five genes affecting the TGFß pathway were induced by heat stress, three of which, SMAD family member 6 (SMAD6), Endoglin (ENG), and TSC22 domain family, member 1 (TSC22D1), modulate signaling by direct interactions with components of the TGFß1 pathway. No changes were detected in the level of TGFß ligands as a function of heat stress. However, only ligands TGFB2 and TGFB3 have been identified in the chicken genome; consequently, our current analysis only quantifies expression of those two genes. The expression data for the TGFß receptors, TGFBR1 and TGFBR2, are conflicting, with the level of TGFBR1 elevated (mean control, 45 RPKM; mean heat-treated, 65 RPKM), but TGFBR2 decreased by heat shock (mean control, 0.13 RPKM; mean heat-treated, undetected). One WNT ligand, WNT4, was increased threefold by heat treatment. An additional four other genes that were elevated by heat stress, Sp5 transcription factor (SP5), secreted frizzled-related protein 1 (SFRP1), dapper, antagonist of beta-catenin (DACT1), and sex determining region Y–box7 (SOX7), inhibit the WNT pathway, either by interfering with intracellular signaling or by blocking canonical WNT-mediated transcription activation (Cavallo et al. 1998; Uren et al. 2000; Takash et al. 2001; Zhang et al. 2006; Fujimura et al. 2007).

The increased expression of SMAD6, KLF6, and CTGF suggests that the TGFß pathway was activated during heat stress. This could be achieved, even in the absence of increased TGFß ligand, by the increase in TGFßR1 receptor expression. Elevated expression of this receptor might increase the sensitivity of cells to existing ligand levels. Increased signaling through the TGFß pathway inhibits apoptosis and improves cell survival during hyperthermia (Jia and Souchelnytskyi 2011). The WNT pathway typically promotes cellular growth. One WNT ligand, WNT4, was upregulated by hyperthermia, and this ligand functions as an antagonist of the canonical WNT signaling pathway. The remainder of the heat-induced genes affecting the WNT pathway typically inhibit WNT signaling. In addition, the chaperone encoded by the *DNAJB6* gene has recently been shown to inhibit the canonical WNT signaling pathway (Mitra et al. 2010), and DNAJB6 was induced in the LMH cells by heat stress. The cumulative effect of heat stress on the TGFß and WNT pathways appears to be promoting cell survival during heat stress by inhibiting apoptosis (TGFß) and suppressing proliferation (WNT inhibition).

### DNA repair and replication

**Fig. 3:**
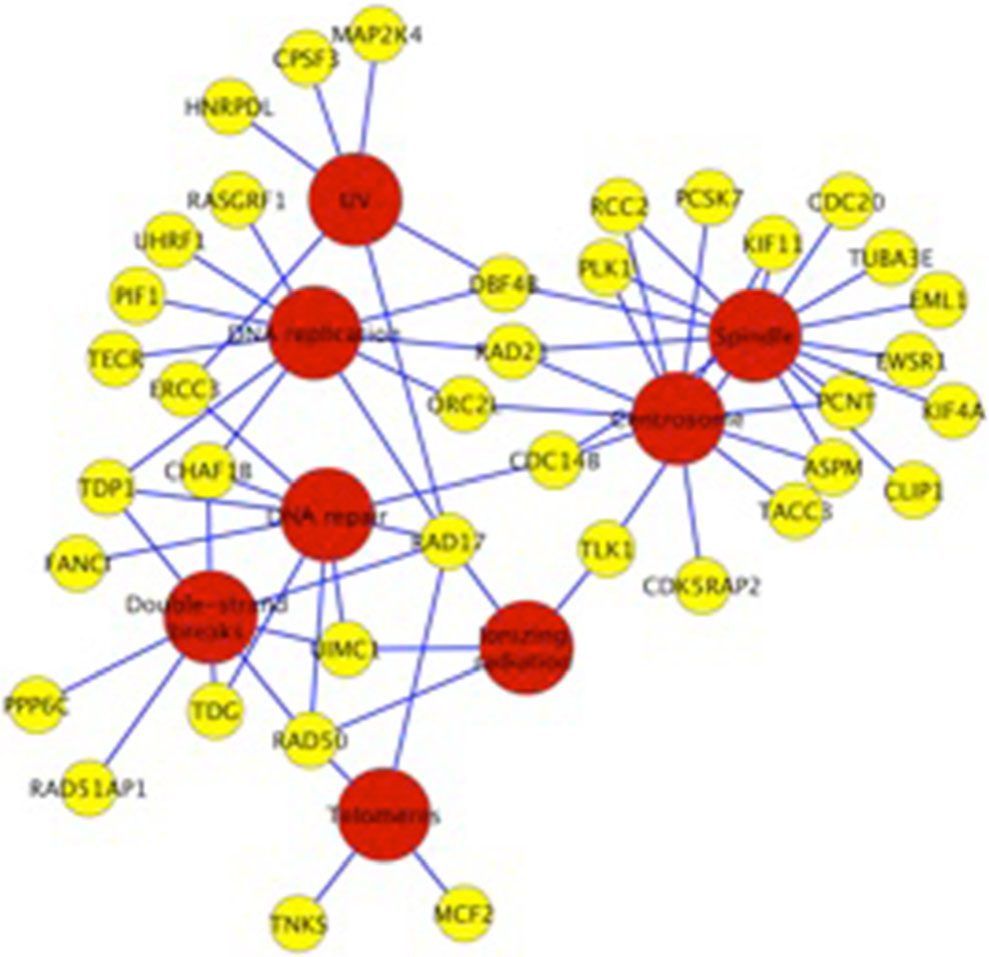
Functional network of selected down-regulated genes. Genes down-regulated by heat stress and associated iTerms affecting DNA replication and repair processes. The *yellow* nodes correspond to gene products and the red nodes refer to iTerms associated with those gene products.

DAVID functional enrichment analysis classified transcripts enriched in control cells into two groups: DNA replication and DNA repair. Characterizing this gene list with eGIFT iTerms supported the DAVID analysis (Table 6 and Fig. 3) and extended it to identify genes involved in chromosomal segregation and telomeric function. Some of these downregulated genes control the onset of S phase, including origin recognition complex, subunit 2 (ORC2), polo-like kinase 1 (PLK1), STE20-like kinase (SLK), and DBF4 homolog B (DBF4B), and decreased expression of these genes may slow the cell cycle. For example, the kinase PLK1 plays an important role in regulating mitotic entry, spindle formation, and cytokinesis (Lenart et al. 2007; Takaki et al. 2008) and PLK1 knockdown in non-transformed diploid cells prolongs S phase (Lei and Erikson 2008).

**Table 6:** iTERMS for genes significantly down-regulated by heat stress

iTERM	Gene symbols
MITOSIS	RASGRF1, TNKS, KIF11, RCC2, RAD21, RAD50 , PCNT, TUBA3E, TACC3, CDC14B, PAPD4, PLK1 TLK1, CDC20, KIF4A, DBF4B, CLIP1, ORC2L, CHAF1B, RAD17, SLK, NEDD9
DNA DAMAGE	RASGRF1, FANCI, RAD21, ERCC3, RAD50, UIMC1 PLK1, PPP6C, DBF4B, TDG, TDP1, CHAF1B, RAD17, UHRF1, CCDC98
MICROTUBULES	CKAP4, KIF11, RAD21, PCNT, TUBA3E, TACC3, GTSE1, PLK1, RANBP10, CDC20, KIF4A, CHFR, CLIP1, SLK
APOPTOSIS	MAP2K4, CKAP4, KIF11, FAF1, BAG1, HIP1R, IKBKB, PLK1, HIP1, SLK, NEDD9
SPINDLE	KIF11, RCC2, RAD21, PCNT, TUBA3E, CDC14B, PLK1, EWSR1, KIF4A, DBF4B, CLIP1
ACTIN CYTOSKELETON	TECR, CLIP1, TUBA3E, SLK, HIP1R, RHOC, MYO6 HIP1, MAGI2, DOCK1
CHROMOSOME	TNKS, RAD21, RAD50, PCNT, ASPM, EWSR1, CDC20, TECR, ORC2L, RAD17
DNA REPLICATION	RASGRF1, RAD21, TECR, DBF4B, ORC2L, TDP1, CHAF1B, UHRF1, RAD17, PIF1
ENDOPLASMIC RETICULUM	LEPRE1, STIM1, RTN1, PTGES2, CYB5R4, PCSK7, MBTPS2, SCFD1, ALG1, ORAI1

This study identified several genes affecting DNA repair and replication that are downregulated by heat stress. The ATR-Chk1 pathway plays a major role in repair of double-stranded breaks (Kampinga and Dikomey 2001; Krawczyk et al. 2011), and one of the several proteins that function in this pathway, Rad17 (Yan and Michael 2009a, 2009b; Tuul et al. 2013) is downregulated in LMH cells by heat stress. Several other proteins affecting DNA repair, DNA replication, spindle formation, chromatin structure, and cell cycle checkpoints were also downregulated by heat stress in the LMH cells (Fig. 3). Given that heat stress responses are frequently conserved across the evolutionary spectrum, these observations in a chicken liver cancer cell line may be relevant to hyperthermic treatment in oncology patients prior to radiation therapy. A major effect of radiation therapy is causing double-stranded breaks in DNA, thereby triggering apoptosis. Downregulating genes that repair such damage might play an important role in sensitizing tumors to radiation treatment (Kaur et al. 2011; Dewhirst and Chi 2013).

### Endoplasmic reticulum and Golgi

eGIFT also identified the endoplasmic reticulum and Golgi as cellular compartments affected by genes downregulated by heat stress. Stromal interaction molecule 1 (STIM1) and ORAI calcium release–activated calcium modulator 1 (ORAI1) gene products are found in the endoplasmic reticulum and both modulate intracellular Ca^2+^ levels. STIM1 is a calcium sensor that activates plasma membrane ORAI1 when calcium levels are depleted in the ER (Putney 2007). This leads to replenishment of the ER calcium stores. Another downregulated gene, membrane-bound transcription factor peptidase, subunit 2 (*MBTPS2*), controls the ER stress response by functioning as a metalloprotease responsible for activating transcription factors, such as activating transcription factor 6 (ATF6), that control the unfolded protein response (Rawson et al. 1997; Haze et al. 1999). While ATF6 expression was detected in the LMH cells, its transcript level did not change in response to heat stress. Three downregulated genes function in protein modification including leucine-proline-enriched proteoglycan 1 (LEPRE1), asparagine-linked glycosylation 1, beta-1,4-mannosyltransferase homolog (ALG1), and ST6 beta-galactosamide alpha-2,6-sialyltranferase 1 (ST6GAL1). LEPRE1 and ALG1 function in the ER, with the former responsible for prolyl hydroxylase activity during collagen maturation (Vranka et al. 2004) while the latter carries out the first step in the production of lipid-linked oligosaccharides (Albright and Robbins 1990). ST6GAL1 is a sialyltransferase that functions in glycosylation in the Golgi (Weinstein et al. 1982, 1987). Finally, three downregulated gene products, sec1 family domain containing 1 (SCFD1), v-SNARE homolog (YKT6), and vacuolar protein sorting 8 homolog (VPS8), function in proper transport of cellular vesicles (Chen and Stevens 1996; Zhang and Hong 2001; Kosodo et al. 2002).

### Translation

Two transcripts downregulated by heat stress, HSPA14 and MTOR associated protein, LST8 homolog (MLST8), have potential roles in controlling translation. HSPA14 encodes a protein that is associated with the ribosome and functions as a chaperone for nascent proteins, controlling their folding as they emerge from the ribosome. Why the expression of HSPA14 is downregulated in LMH cells during heat stress is uncertain. Potentially, reduction of HSPA14 could lower the rate of translation by slowing the emergence of properly folded proteins from the ribosome. This could contribute to reducing the protein synthesis burden during heat stress. MLST8 is a component of the mTORC1 complex, and one effect of mTORC1 activation is increased translation. Reduction of MLST8 levels would likely reduce overall protein synthesis by lowering the available pool of mTORC1. Taken together, downregulation of these two transcripts may play a direct role in the overall repression of protein synthesis seen during heat stress (Yamasaki and Anderson 2008; Shalgi et al. 2013)

### Correlation analysis

**Fig. 4:**
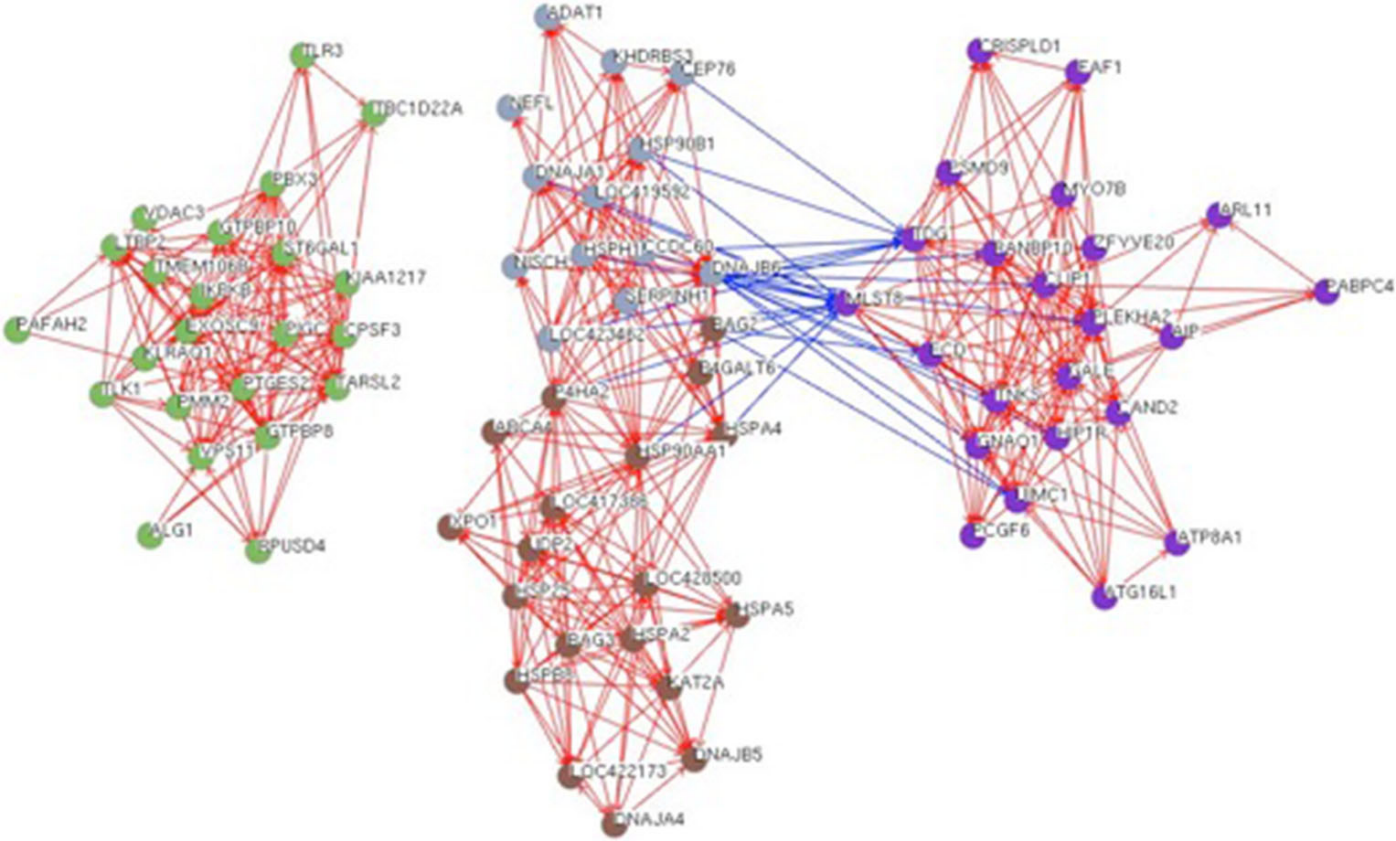
Pearson Correlation Network Networks containing significantly correlated gene pairs (Pearson correlation coefficient > 0.95) that were modulated either up or down by heat stress. Nodes in cluster 1 (*green*) or cluster 2 (*purple*) were down-regulated in heat challenge while nodes in cluster 3 (*gray*) or cluster 4 (*brown*) were up-regulated. Red edges indicate positive correlation between gene pairs while blue edges indicate negative correlation.

Pearson correlation analysis was conducted on all differentially expressed genes and identified four networks of heat-modulated genes (Fig. 4). We limited this analysis to genes having connections to at least three other genes with correlation coefficients greater than 0.95 (Mansson et al. 2004). Two of the clusters contained downregulated genes (clusters 1 and 2) while the other two (clusters 3 and 4) contained upregulated genes. Highly correlated downregulated genes affected a variety of processes including DNA replication and repair, mRNA polyadenylation, pro-inflammatory responses, and glycosylation. The cluster of upregulated genes is predominantly comprised of genes encoding molecular chaperones along with some genes not previously associated with heat stress response. Finally, clusters 2, 3, and 4 are connected by several downregulated genes in cluster 2 that show strong negative correlation with several genes that are upregulated by heat stress.

A reasonable hypothesis is that individual clusters contain genes that are coordinately regulated by the same transcription factors. Given that HSFs form functional heterotrimers (Sandqvist et al. 2009), it is possible that different combinations of the HSFs have distinct transcription factor activities and could yield these different networks. Also, the strong negative correlations between up regulated and downregulated genes (i.e., HSPA4 and MLST8) in clusters 2, 3, and 4 suggest a mechanism in which the same transcription factor is positively regulating one set of genes (clusters 3 and 4), while negatively regulating another set (cluster 2). Future work can use overexpression and knockdown approaches to evaluate the impact of changing the levels of different transcription factors on these networks.

It is possible that heat responsive genes may function in setting body temperature. The Animal Quantitative Trait Loci (QTL) Database (Hu et al. 2013)(Release 24, Aug 25, 2014) identifies QTLs associated with body temperature mapped to five chicken chromosomes (chromosomes 2, 3, 4, 6, and 23). Inspecting the chromosomal locations of the heat stress responsive genes defined in this study (Table 7) provides a total of 25 genes located within the QTL boundaries on chromosomes 2, 3, 4, and 6. These 25 can be considered candidate genes for regulating body temperature.

**Table 7:** Heat Responsive genes mapping to QTL locations that regulate body temperature. QTL locations were obtained from the Animal QTL Database

GENE	CHROMOSOME	Start	Finish	Ave. Control RPKM	Ave. Heat Stress RPKM
NSUN2	2	82,300,000	87,200,000	93.5	49.4
MOCOS	2	82,300,000	87,200,000	2.01	1.08
PDE10A	3	35,500,000	51,300,000	12.5	28.5
PNLDC1	3	35,500,000	51,300,000	0.09	0.24
SHPRH	3	35,500,000	51,300,000	1.65	0.87
UST	3	35,500,000	51,300,000	3.80	7.25
EXOSC9	4	50,900,000	88,400,000	35.7	12.6
SYNPO2	4	50,900,000	88,400,000	0.35	0.13
METTL14	4	50,900,000	88,400,000	24.4	13.5
TLR3	4	50,900,000	88,400,000	3.28	1.64
SLC7A2	4	50,900,000	88,400,000	0.89	0.34
KCTD8	4	50,900,000	88,400,000	4.00	1.25
ATP8A1	4	50,900,000	88,400,000	3.66	1.80
RBM47	4	50,900,000	88,400,000	28.9	16.5
CHRNA9	4	50,900,000	88,400,000	0.33	0.08
PCDH7	4	50,900,000	88,400,000	0.88	0.44
ANAPC4	4	50,900,000	88,400,000	7.65	4.23
CCDC149	4	50,900,000	88,400,000	4.84	2.11
TBC1D14	4	50,900,000	88,400,000	14.3	7.18
AFAP1	4	50,900,000	88,400,000	3.70	8.21
TACC3	4	50,900,000	88,400,000	78.7	29.6
LOC423899	6	4,500,000	33,800,000	0.26	0.07
BAG3	6	4,500,000	33,800,000	33.8	806.0
LHPP	6	4,500,000	33,800,000	11.1	6.15
DOCK1	6	4,500,000	33,800,000	6.11	2.65

Overall, this work has identified 812 genes whose transcription is modified by heat stress. Based on numerous previous studies, many of the genes identified in the current study were predicted to be responsive to heat stress (Lindquist and Craig 1988; Akerfelt et al. 2010). However, several of the genes either upregulated or downregulated by heat challenge in our studies have not, to our knowledge, been previously identified as heat responsive. Multiple biological processes were affected by the responsive genes including translation, transcription, chromatin modification, DNA repair, and DNA synthesis. In addition, two signaling pathways were modulated by heat stress: TGFß and WNT. The heat responsive genes affecting the TGFß pathway indicate activation of this pathway, while the WNT pathway appears to be inactivated. Current studies in our laboratory are using RNA-seq to identify heat responsive genes following hyperthermic treatment of chickens with the goal of providing a more complete catalog of heat responsive genes.

### Broader implications

We have examined the transcriptome response of the chicken LMH hepatocarcinoma cell line to hyperthermia using RNA-seq and identified biological processes and pathways impacted by heat stress. It is important to recognize the nature of the cells studied; the LMH cell line was obtained from a male chicken fed diethylnitrosamine, a potent inducer of liver cancer (Kawaguchi et al. 1987). The line was derived in 1987 and has adapted to long-term growth in cell culture. When comparing our results with analyses of response to heat stress in other cell lines or tissues, both similarities and differences are noted. Among the major similarities across cell lines from different species are increases in transcripts encoding chaperones and co-chaperones that play important roles in maintaining homeostasis during heat stress (Lindquist and Craig 1988; Murray et al. 2004). Responses in these functional groups of genes are also noted in the livers of chickens (Schmidt, unpublished), ducks (Zeng et al. 2014), and other animals (Flanagan et al. 1995). Another common response to heat stress are changes in gene expression consistent with cell cycle arrest (Sonna et al. 2002) although this is variable. For example, the human fibroblasts exhibit cell cycle arrest during hyperthermia while human Hela and K-562 cancer cell lines do not (Murray et al. 2004). Upregulation of the TGFß pathway through different mechanisms has been noted in other cell culture systems (Jia and Souchelnytskyi 2011; Li et al. 2015) and in scrotal tissues (Cai et al. 2011) during heat stress. In reviewing this information, it becomes apparent that not all cell lines or tissues respond to heat stress in exactly the same fashion. The total response likely depends on a combination of variables including the genetic composition of the target species, the genomic changes that have occurred due to past environmental stresses, and the nature of the current heat challenge (Sonna et al. 2002). An important future goal will be to relate heat stress transcriptome responses at the cellular and organismal level to these different variables.

### Electronic supplementary material

Supplementary File 1 Genes analyzed Complete comma delimited file containing all genes analyzed in this study along with RPKM values for each gene in each experiment. (CSV 1563 kb)

Supplementary File 2 812 genes differentially regulated by heat stress. Comma delimited file of all genes that were differentially regulated by heat stress with a P value less than 0.01. (CSV 83 kb)

Supplementary File 3 Principal component anlaysis A PNG formated file of principal component analysis of the 812 genes differetnially regualted by heat stress (JPEG 36.6 kb)

Supplementary File 4 A complete comma delimited list of symbols and names for all genes discussed in ths paper. (CSV 6 kb)

## Appendix

The data sets supporting the results of this article are included within the article and its additional files
