# Supplementary figures and images for: Transcriptome response to heat stress in a chicken hepatocellular carcinoma cell line
Source: Cell Stress Chaperones. 2015 Aug 5;20(6):939–50. doi: 10.1007/s12192-015-0621-0 (PMC4595433; doi:10.1007/s12192-015-0621-0)

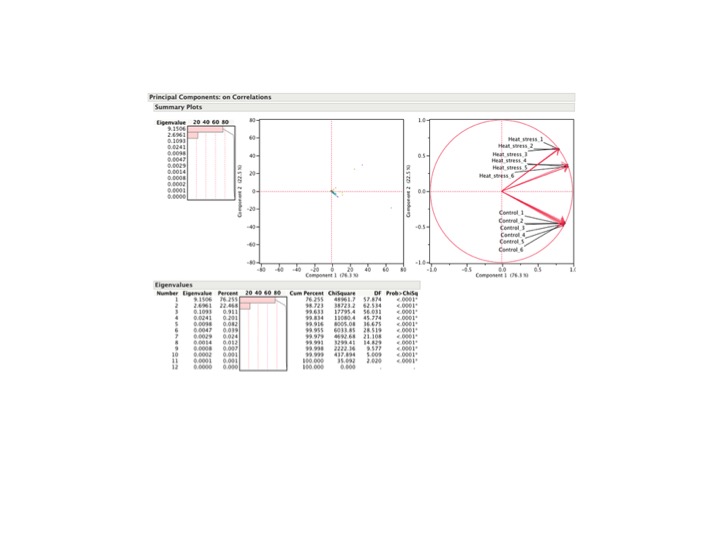

Supplement: Supplementary file 3 — Principal component anlaysis A PNG formated file of principal component analysis of the 812 genes differetnially regualted by heat stress (JPEG 36.6 kb) [file 12192_2015_621_MOESM3_ESM.jpg]
